# Proinflammatory response of canine trophoblasts to *Brucella canis* infection

**DOI:** 10.1371/journal.pone.0186561

**Published:** 2017-10-16

**Authors:** Andrea G. Fernández, M. Soledad Hielpos, Mariana C. Ferrero, Carlos A. Fossati, Pablo C. Baldi

**Affiliations:** 1 Universidad de Buenos Aires, Facultad de Farmacia y Bioquímica, Cátedra de Inmunología, Buenos Aires, Argentina; 2 CONICET-Universidad de Buenos Aires, Instituto de Estudios de la Inmunidad Humoral (IDEHU), Buenos Aires, Argentina; 3 Instituto de Estudios Inmunológicos y Fisiopatológicos (IIFP, CONICET-UNLP), Facultad de Ciencias Exactas, Universidad Nacional de La Plata, La Plata, Argentina; Otto von Guericke Universitat Magdeburg, GERMANY

## Abstract

*Brucella canis* infection is an important cause of late-term abortion in pregnant bitches. The pathophysiological mechanisms leading to *B*. *canis*–induced abortion are unknown, but heavily infected trophoblasts are consistently observed. As trophoblasts responses to other pathogens contribute to placental inflammation leading to abortion, the aim of the present study was to characterize the cytokine response of canine trophoblasts to *B*. *canis* infection. To achieve this, trophoblasts isolated from term placenta of healthy female dogs were infected with *B*. *canis*, culture supernatants were harvested for cytokine determinations, and the load of intracellular viable *B*. *canis* was determined at different times post-infection. Additionally, cytokine responses were assessed in non-infected trophoblasts stimulated with conditioned media (CM) from *B*. *canis*-infected canine monocytes and neutrophils. Finally, cytokine response and bacteria replication were assessed in canine placental explants infected *ex vivo*. *B*. *canis* successfully infected and replicated in primary canine trophoblasts, eliciting an increase in IL-8 and RANTES (CCL5) secretion. Moreover, the stimulation of trophoblasts with CM from *B*. *canis*-infected monocytes and neutrophils induced a significant increase in IL-8, IL-6 and RANTES secretion. *B*. *canis* replication was confirmed in infected placental explants and the infection elicited an increased secretion of TNF-α, IL-8, IL-6 and RANTES. This study shows that canine trophoblasts produce proinflammatory cytokines in response to *B*. *canis* infection and/or to stimulation with factors produced by infected monocytes and neutrophils. These cytokines may contribute to placental inflammation leading to abortion in *B*. *canis*-infected pregnant bitches.

## Introduction

Brucellosis is a worldwide distributed zoonosis caused by Gram negative bacteria of the *Brucella* genus [1]. Whereas most cases of human brucellosis are related to contact with secretions or tissues from livestock (cows, pigs, goats, sheep), there have been increasing reports of brucellosis acquired from infected dogs [2–4]. The vast majority of cases of canine brucellosis are caused by *B*. *canis*, although occasional infections due to other *Brucella* species have been reported. The most prominent clinical consequences of *B*. *canis* infection in dogs relate to genital organs, including late-term abortion and fetal resorption in pregnant females, and orchiepididymitis and prostatitis in males [5,6]. The bacterial load of *B*. *canis* is extremely high in vaginal secretions of infected bitches during estrus and parturition, and also in fetal and placental tissues from abortions [5]. In addition, infected males excrete bacteria in their semen. Healthy dogs can acquire the infection through genital, oronasal or conjuctival mucosae. Therefore, transmission occurs easily between dogs in close contact, especially in kennels where a high proportion of dogs is usually affected resulting in important economic losses due to abortions and infertility [7–9].

It has been consistently shown that trophoblasts are a main target of *B*. *canis* infection in pregnant bitches, and heavily infected trophoblasts have been repeatedly observed in aborted canine placentas [5,8,9]. However, the exact pathophysiological mechanisms leading to abortion due to *B*. *canis* have not been described. Nevertheless, histological studies reveal infiltrates of neutrophils and mononuclear cells in the aborted placentas, suggesting that local inflammation may play a role [5,9]. Notably, in pregnant cattle *B*. *abortus* replication within trophoblasts induces the infiltration of inflammatory cells, trophoblast necrosis, vasculitis, and ulceration of the chorioalantoid membrane, finally leading to abortion [10,11].

Inflammatory responses in placental tissues have been related to infection-triggered abortion, and in many cases trophoblasts have been shown to contribute to such inflammation [12–16]. In line with these findings, the *in vitro* stimulation of human trophoblasts with bacterial or viral pathogens, or their antigens, induces an increased secretion of several cytokines and chemokines including IL-1β, IL-6, TNF-α, IL-8 and MCP-1 [17–21]. To our best knowledge, similar studies have not been performed with canine trophoblasts.

While histological studies indicate that trophoblasts are a main target of *B*. *canis* infection in pregnant bitches, the cytokine response of canine trophoblasts to such infection has not been explored. In the present study, we employed primary canine trophoblasts and placental explants to address this issue.

## Materials and methods

### Bacterial strains and growth conditions

The RM6/66 strain of *Brucella canis* was provided by the Brucella Reference Laboratory at the National Institute for Infectious Diseases (INEI, ANLIS-Malbrán, Buenos Aires). Bacteria were grown overnight in tryptic soy broth (TSB), harvested by centrifugation, and washed twice in phosphate-buffered saline (PBS). Bacterial numbers in cultures were estimated by comparing the OD at 600 nm with a standard curve, but the actual concentration of inocula was checked by plating on tryptic soy agar plates (TSA). All live *Brucella* manipulations were performed in biosafety level 3 facilities.

### Canine placenta and blood collection

The dogs used for this study were all healthy pets fed by their owners with fresh water and balanced food according to the veterinarians’ advice. Owners gave their formal consent to provide the sample, either placenta or blood. The protocols for obtaining canine placentas, monocytes and neutrophils (see below) were approved by the Institutional Committee for the Care of Laboratory Animals (protocol N° 18112015–73).

Blood was obtained from 4 dogs by puncture of the antebrachial cephalic vein. After hair removal and skin antisepsis with iodopovidone, 15 ml of blood was collected in a sterile syringe. The blood was transferred immediately to a 50-ml sterile tube containing 50 μl of sodium heparin 50.000 UI (Northia®), and transported refrigerated to the laboratory where it was processed immediately.

Thirty-three dog term placentas were obtained by C-section, routinely performed in a veterinary practice, from healthy female pregnant bitches; the surgical procedure is described below. First, hair was removed from the lower back, one of the forearms and abdomen; those areas were washed with a chlorhexidine gluconate 4% solution and rinsed afterwards. Each dog was taken to the operating room, where an intravenous teflon catheter of 20 to 24 gauge was placed according to the size of the animal, and was connected to a set for saline infusion, which was administered by gravity drip during the entire surgical procedure. A mask was also placed for the administration of oxygen throughout the surgery. Pre-anesthesia was given through the infusion set and antisepsis of the lumbar area was performed using a chlorhexidine solution, applied and removed 3 consecutive times. Afterwards, epidural anesthesia was administered to provide greater safety to the fetus and the mother. The patient was placed in dorsal decubitus position and antisepsis was performed as described above. The sterile field cloths were placed. An incision was made in the pubic sternal skin, divulsion of the subcutaneous tissues was performed, and the abdominal muscles were incised by the alba line. The uterus was incised, the fetal wraps were torn and each fetus was removed by pinching its umbilical cord with a hemostatic clamp. Each placenta was then drawn and placed in a sterile container. The uterus, the abdominal wall, the subcutaneous and the skin were sutured. The placentas were preserved refrigerated for no more than 24 h in Dulbecco modified Eagle medium with antibiotics and transported to the lab for processing. Once the surgery was completed the good health of the patient and its puppies was verified by cardiac and respiratory auscultation and control of mucosal coloration. They were sent home with their owners who administered analgesics and antibiotics. At 15 days, unless they required a previous consultation, they returned to the clinic for removal of stitches and deworming and control of the puppies.

None of the methods of sample collection ended with the sacrifice of the dogs.

### Canine trophoblasts isolation

Canine trophoblastic cells were isolated from 30 term placenta obtained by C-section of healthy female dogs following previously described procedures [22]. Briefly, after removing the extra-chorionic membranes and marginal hematoma, the villous chorioallantois was cut into 0.5 cm pieces, washed thoroughly with PBS to eliminate blood, and incubated with a collagenase solution at 37°C for 10 min. After sedimentation on an ice bath, the supernatant was aspirated and discarded and the remaining tissue was incubated with a trypsin solution for a further 10 minutes. After sedimentation, the supernatant was collected and mixed with wash media containing fetal calf serum (FCS) to halt enzymatic digestion. Next, samples were centrifuged and the cell pellet was treated with collagenase for approximately 3 minutes. After centrifugation at low speed the cell pellet was resuspended in cell culture media and added to the top of a Percoll (GE Healthcare) gradient (20–70%). The samples were centrifuged at 1573 xg for 25 minutes at 4°C, and the top two bands of the Percoll gradient were collected. After several washing steps the cell pellet was resuspended in Dulbecco modified Eagle medium: Nutrient mixture F12 (HAM) (1:1) (DMEM/ F12) (Gibco-BRL Life Technologies) supplemented with 2 mM L-glutamine (Gibco), 1% FCS (Gibco), 100 U/ml penicillin, 25 μg/ml of gentamicin (Sigma) and 100 μg/ml streptomycin (Sigma) (complete medium) and distributed on culture flasks or multiwell plates. Cell viability was >98%, as determined by trypan blue exclusion. After several days in culture in a 5% CO_2_ atmosphere at 37°C, the purity of the primary trophoblasts was verified by indirect immunofluorescence with an anti-cytokeratin 7 antibody (BD Biosciences). Previous studies have shown that among cells from canine placenta only trophoblasts stain positive for cytokeratin-7 [22]. Fibroblast contamination was evaluated by immunofluorescence with an anti-vimentin antibody (Sigma). A potential leukocyte contamination of the isolated cells was evaluated in a BD FACSCalibur flow cytometer with a FITC-labeled anti-dog CD45 monoclonal antibody (AbDSerotec).

### Stimulation with Toll-like receptor agonists and *Brucella* antigens

Canine trophoblasts were seeded at 5 × 10^4^ cells/ml in 24-well plates in complete medium. Cells were stimulated with 5μg/ml of Pam3CSK4 (InvivoGen), 5 μg/ml of *E*. *coli* lipopolysaccharide (Sigma), 1 μg/ml of flagellin from *Salmonella enterica* serovar Typhimurium (kindly provided by Martín Rumbo, IIFP, Argentina) or 10^9^ colony forming units (CFU) /ml of heat-killed *Brucella canis* (HKBc). Culture supernatants were harvested after 24h to measure chemokines and cytokines.

### Isolation of canine monocytes and neutrophils

Canine monocytes and neutrophils were isolated from venous blood of 4 healthy dogs by Ficoll-Paque (GE Healthcare) gradient (the procedure for blood collection was described above). To isolate monocytes, the buffy coat was harvested, the cells were resupended in RPMI 1640 medium, and seeded at 2 × 10^6^ cells/ml in 24-well plate for 2h to achieve adherence. Afterwards, cells were washed with PBS to discard non-adherent cells and supplemented with RPMI medium with 5% FCS. To isolate neutrophils, the pellet obtained after Ficoll gradient centrifugation (containing erythrocytes and granulocytes) was mixed with 6% dextran to allow red cells sedimentation. Neutrophils were harvested and, after hypotonic lysis of remaining erythrocytes, were washed twice with PBS, resuspended in RPMI 1640 supplemented with 5% FCS, and 1 mM L-glutamine, and seeded at 5 × 10^5^ cells/ml in 24-well plates. Cell viability was >98%, as determined by trypan blue exclusion. The purity of the final neutrophil preparation was >95% as determined by morphological examination with Giemsa staining.

### Cellular infections

Canine trophoblasts were infected with *B*. *canis* at a multiplicity of infection (MOI) of 250 bacteria/cell, whereas canine monocytes and neutrophils were infected at MOI 100. For all infections cells were dispensed at appropriate densities in 24 well-plates. After dispensing the bacterial suspension, the culture plates were centrifuged (10 min at 300 x*g* at room temperature) and then incubated for 2 hours at 37°C under 5% CO_2_ atmosphere. At the end of the incubation time, each well was washed three times with sterile PBS. To kill extracellular bacteria, cell cultures were incubated with 100 μg/ml of gentamicin (Sigma) and 50 μg/ml of streptomycin (Sigma). At different times after antibiotics addition (2, 24 or 48 h p.i.) culture supernatants were harvested for cytokine determinations. In the case of trophoblasts infection, in addition, cells were washed with sterile PBS and lysed with 0.2% Triton X-100, and serial dilutions of the lysates were plated on TSA to enumerate CFU.

### Cellular stimulation with conditioned media

Canine monocytes and neutrophils were infected with *B*. *canis* at a MOI of 100 for 2 h, washed with sterile PBS, and incubated with antibiotics to kill extracellular bacteria. Conditioned media (CM) from infected cells were harvested at 24 h p.i., sterilized by filtration through 0.22 μm nitrocellulose filters, and used to stimulate uninfected canine trophoblasts. The absence of live bacteria in CM was routinely tested by plating on bacteriological agar and incubating at 37°C for 7 days. As a control, trophoblasts were also stimulated with CM from uninfected phagocytes incubated for 24 h under the same conditions. For stimulation, CM were used diluted 1/5 in complete medium. At 24 h of stimulation, the supernatants from stimulated cultures were harvested to measure cytokines and chemokines. To calculate the specific secretion of each factor, levels already present in CM from phagocytes were subtracted from levels measured after stimulation.

### Canine placental explants culture and infection

Three term placentas were obtained by C-section of healthy pregnant bitches (as described above) and preserved refrigerated for less than 24 h in DMEM/F12 supplemented with Ceftazidime (50 μg/ml). After the removal of the extra-chorionic membranes and marginal hematoma, explants from the villous chorioallantois were obtained using an 8 mm diameter biopsy punch, and were cultured in 24 well-plates in complete medium without antibiotics. To infect the explants, 1x10^8^ CFU of *B*. *canis* were added to each well, the plates were centrifuged for 10 min at 300 x*g* at room temperature, and were incubated for 2 h at 37°C under 5% CO_2_ atmosphere. The explants were later washed three times with PBS and extracellular bacteria were killed by incubation with culture medium containing 100 μg/ml of gentamicin and 50 μg/ml of streptomycin for 2h. Afterwards, antibiotics were removed and explants culture was continued up to 48 h p.i. At 2, 24 and 48 h p.i. culture supernatants were collected to measure cytokines and chemokines. Explants homogenates were manually performed in 1 ml of sterile PBS using a Potter-Elvehjem tissue homogenizer, and were plated on TSA to count CFU.

### Cytokine and chemokine measurement

Canine IL-6, IL-8, TNF-α and RANTES (CCL5) were measured in culture supernatants from *B*. *canis-*infected cells or in culture supernatants from trophoblasts stimulated with CM from *B*. *canis*-infected phagocytes by sandwich ELISA (all from R&D, Minneapolis, USA) using paired cytokine-specific mAbs, according to the manufacturer’s instructions.

### Cytotoxicity assay

To evaluate whether *B*. *canis* infection generates cell membrane damage in canine trophoblasts, the release of lactate dehydrogenase (LDH) to the culture medium was determined. Culture supernatants from trophoblasts infected with *B*. *canis* at MOI 250 for 24 and 48 h were collected, and LDH activity was measured using CytoTox 96 non-radioactive cytotoxicity assay (Promega). Results are expressed in arbitrary units.

### Statistical analysis

Statistical analysis was performed with Student᾽s *t* test, Mann-Whitney test or with one-way ANOVA followed by Tukey᾿s test or Dunnett’s test using GraphPad Prism 5.0 software. Values are represented as mean ± SD.

## Results

### Primary canine trophoblasts isolation and immune response to TLR agonists and *B*. *canis* antigens

**Fig 1:**
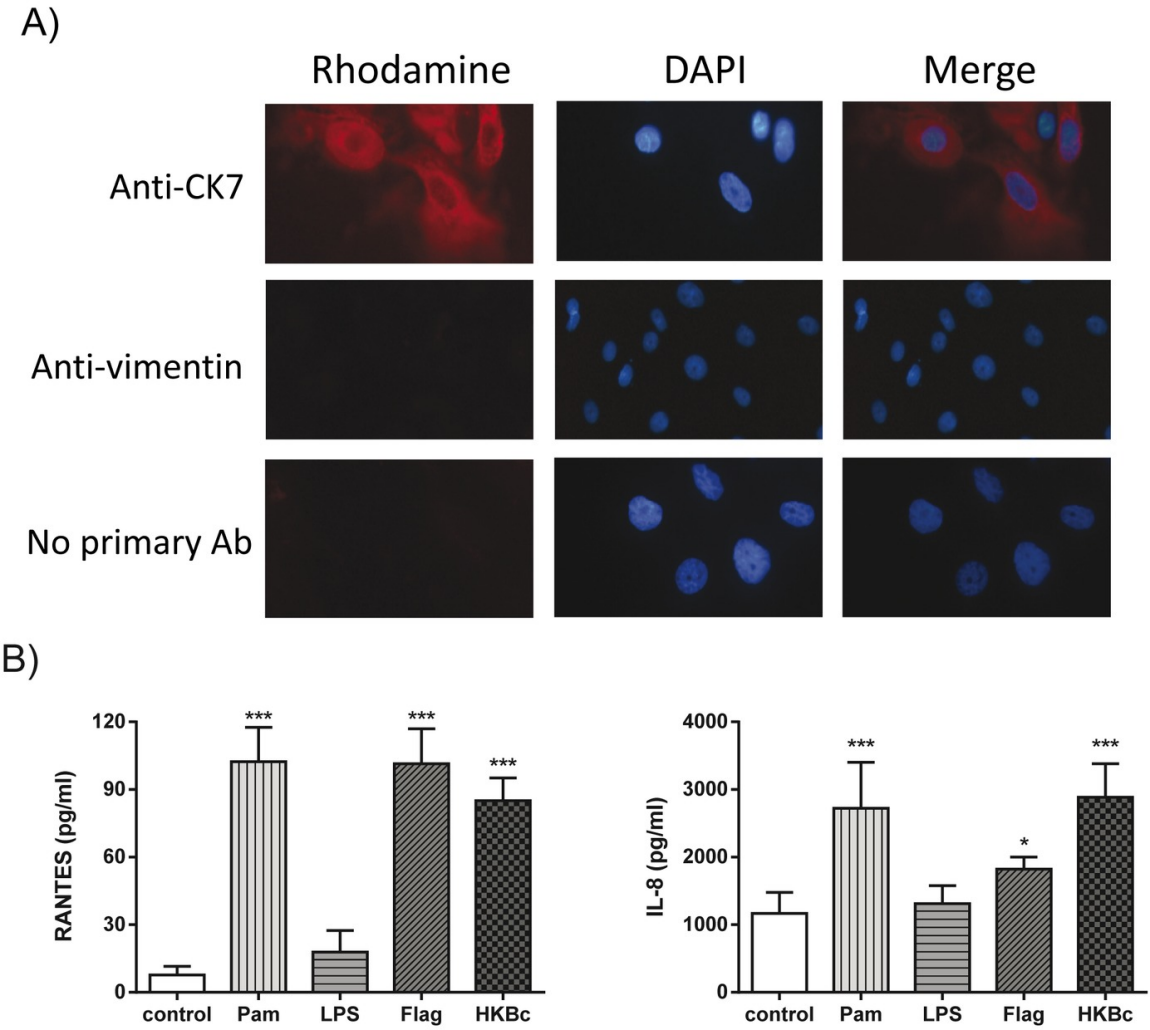
Isolation of canine trophoblasts and chemokine response to stimulation with TLR agonists and *B*. *canis* antigens. (A) Trophoblasts isolated from term placentas of healthy dogs stained positive for cytokeratin 7 and negative for vimentin. (B) Chemokine response of canine trophoblasts isolated from 6 term canine placentas stimulated for 24 h with 5 μg/ml Pam3CSK4 (Pam), 5 μg/ml of *E*. *coli* lipopolysaccharide (LPS), 1 μg/ml of *S*. *enterica* flagellin (Flag) and 10^9^ colony forming units (CFU) /mL of heat-killed *Brucella canis* (HKBc). Values in B are means ± SD of duplicate measurements from three independent experiments. Asterisks indicate significant differences as compared to unstimulated (control) cells (* p<0.05; *** p<0.001, ANOVA followed by Dunnett᾽s test).

Canine trophoblastic cells were isolated from term placenta of healthy female dogs following established procedures. The identity of the cells as trophoblasts was confirmed by their positive staining for cytokeratin-7 and by their negative staining for vimentin, which is highly expressed in canine fibroblasts but not in trophoblasts [22] (Fig 1A). For each isolation procedure, at least 100 DAPI-positive cells were evaluated by immunofluorescence for cytokeratin 7 and vimentin staining. All the cell suspensions analyzed had more than 97% cytokeratin 7-positive cells and less than 3% vimentin-positive cells. Leukocyte contamination of trophoblastic cells was <1% as determined by flow cytometry (S1 Fig).

To assess the functionality of the isolated canine trophoblasts in terms of innate immunity, the cytokine response of these cells to stimulation with TLR agonists or HKBc was measured. As the TLR repertoire of canine trophoblasts has not been reported, agonists for surface TLRs known to induce cytokine secretion in human trophoblasts were used [23,24]. As shown in Fig 1B, the secretion of RANTES (CCL5) and IL-8 was significantly increased in canine trophoblasts upon stimulation with agonists for TLR1/2 (Pam3CK4) and TLR5 (flagellin) while there was no increase in chemokine levels upon stimulation with a TLR4 ligand (*E*. *coli* LPS). Furthermore, the secretion of both chemokines also increased upon stimulation with HKBc. No secretion of IL-6 or TNF-α was detected in culture supernatants of either stimulated or unstimulated cells.

### *Brucella canis* infection of canine trophoblasts and chemokine response

**Fig 2:**
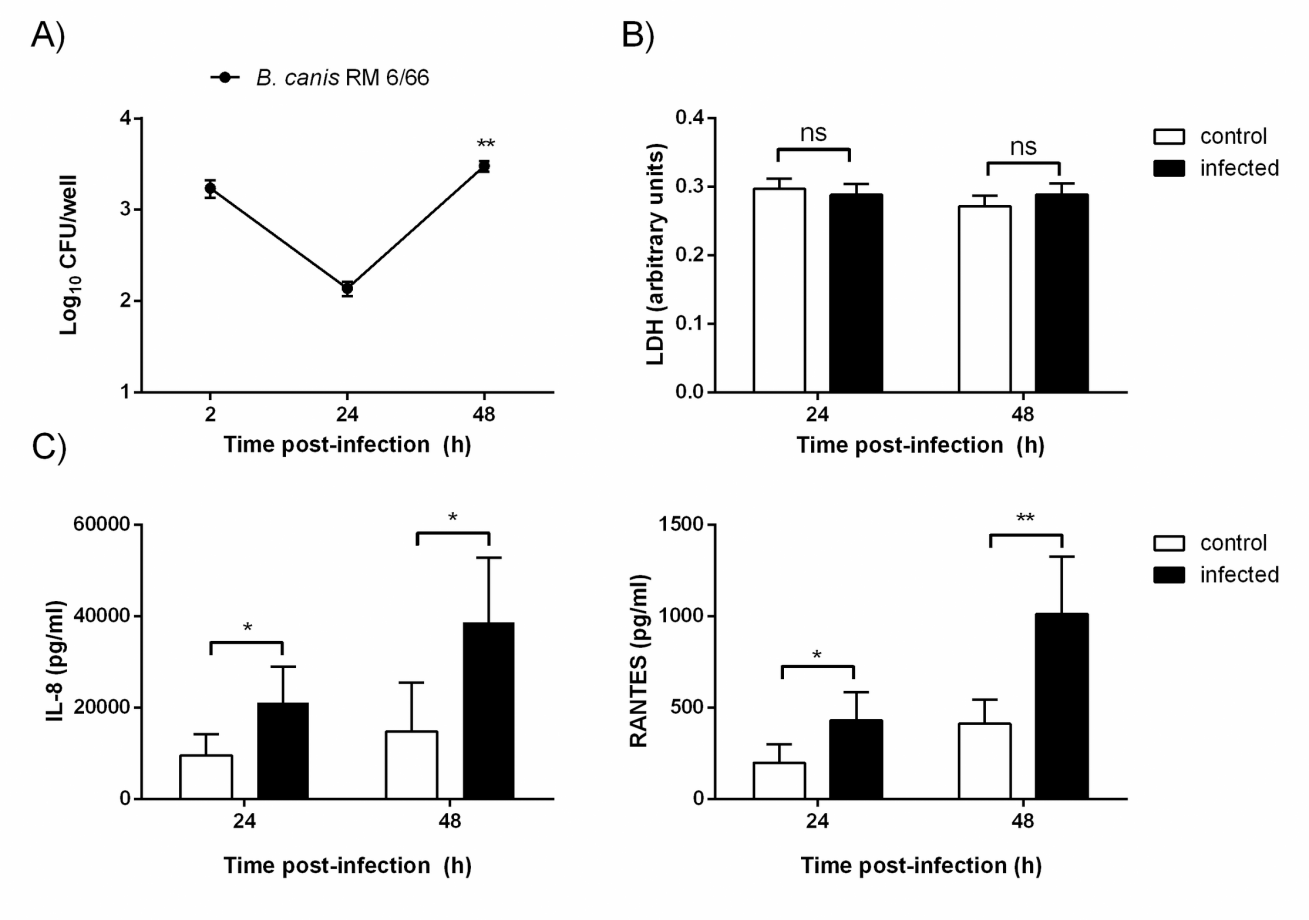
*B*. *canis* infects and replicates in canine trophoblasts without inducing cytotoxicity, but inducing proinflammatory chemokines. Trophoblasts isolated from 6 term placentas (1x10^5^cells) were infected with *B*. *canis* at MOI 250 for up to 48 h. (A) After addition of antibiotics to kill extracellular bacteria, cells were lysed at 2, 24 and 48h to enumerate CFU counts. (B) Culture supernatants from trophoblasts uninfected and infected with *B*. *canis* were harvested at 24 and 48 h p.i. to measure lactate dehydrogenase (LDH) activity. Values in A and B are means ± SD of duplicate measurements from three independent experiments. Asterisks indicate significant differences between 24 and 48h p.i (** p<0.01, Mann-Whitney test). (C) Culture supernatants were harvested at 24 and 48 h p.i. to measure IL-8 and RANTES (CCL5) secretion. Culture supernatants from uninfected trophoblasts cultured in parallel under the same conditions were harvested as controls. Values are means ± SD of duplicate measurements from three independent experiments. Asterisks indicate significant differences as compared to uninfected (control) cells (* p<0.05; ** p<0.01, Student᾽s *t* test).

Trophoblasts were infected with *B*. *canis*, treated with antibiotics to kill extracellular bacteria, and lysed at different times p.i. to determine CFU counts of intracellular bacteria. As shown in Fig 2A, after an initial decrease of viable bacteria, *B*. *canis* was able to replicate in canine trophoblasts, with a 22-fold increase of intracellular CFU between 24 and 48 h pi. The infection did not seem to affect the viability of trophoblasts as levels of LDH activity in culture supernatants did not differ between infected and uninfected cells at either 24 or 48 h p.i. (Fig 2B).

Culture supernatants from trophoblasts infected with *B*. *canis* were harvested at 24 and 48 h p.i. to measure cytokine and chemokine levels. As shown in Fig 2C, at both time points levels of RANTES and IL-8 were significantly higher in infected cells than in uninfected cells, indicating an infection-specific secretion of both chemokines. IL-6 and TNF-α could not be detected in culture supernatants of either infected or uninfected cells.

### Chemokine response of trophoblasts to factors produced by *B*. *canis*-infected phagocytes

**Fig 3:**
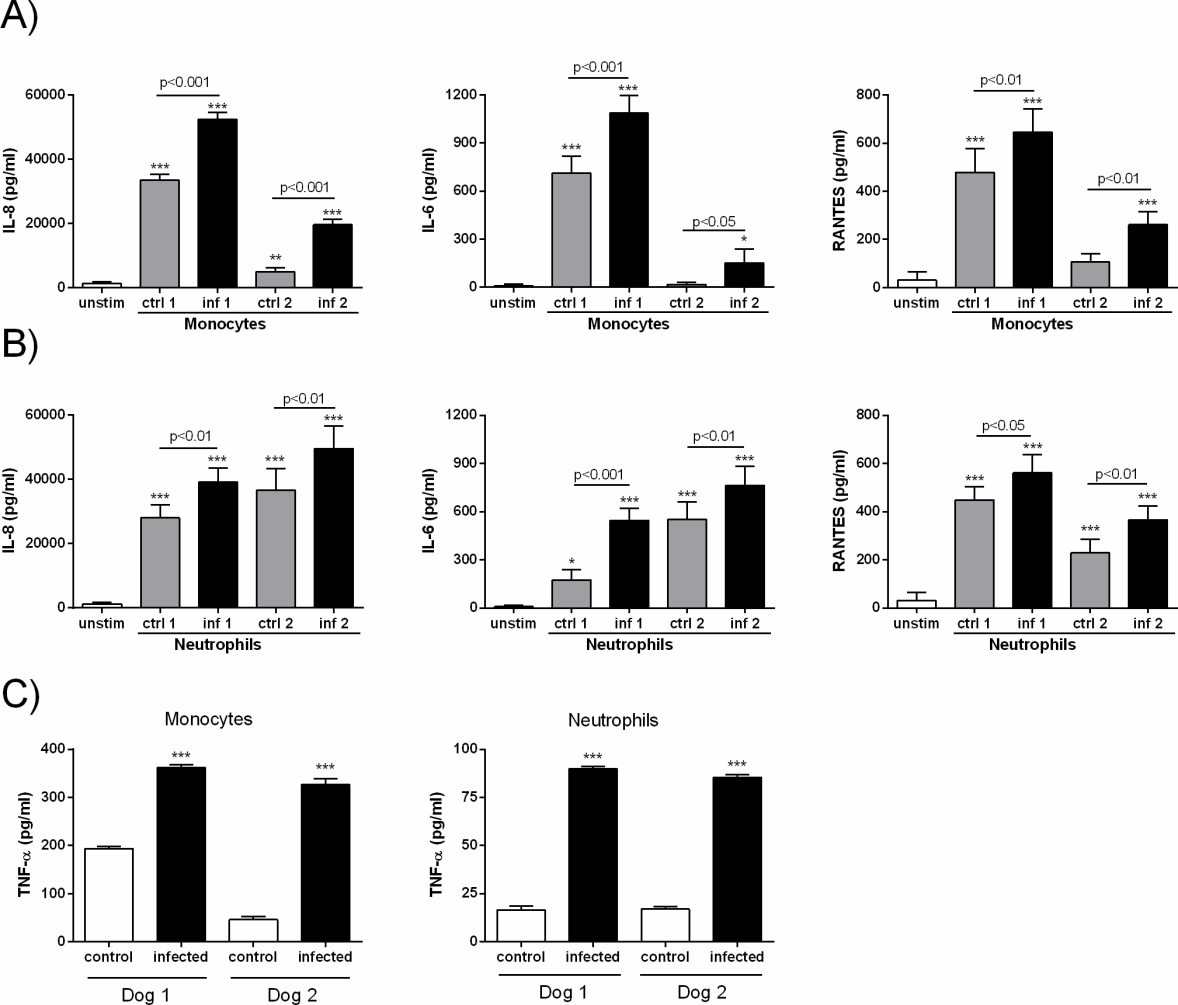
Canine trophoblasts secrete proinflammatory chemokines and cytokines in response to stimulation with conditioned media (CM) from *B*. *canis*-infected monocytes and neutrophils. Trophoblasts isolated from 12 term placentas were stimulated for 24 h with 1/5 dilutions of the CM. Unstimulated trophoblasts cultured under the same conditions served as controls. Levels of IL-8, IL-6 and RANTES (CCL5) were measured in culture supernatants of trophoblasts stimulated with CM from monocytes (A) or neutrophils (B). Values are means ± SD of duplicate measurements from three independent experiments. Asterisks over bars indicate significant differences as compared to unstimulated trophoblasts (* p<0.05; ** p<0.01; *** p<0.001, ANOVA followed by Tukey᾿s test). C) TNF-α secretion by *B*. *canis*-infected canine monocytes and neutrophils. The cells were purified from peripheral blood of 4 healthy dogs, and were infected *in vitro* with *B*. *canis* (MOI 100). TNF-α was measured in culture supernatants at 24 h p.i. Values are means ± SEM of triplicate measurements. Asterisks indicate significant differences as compared to uninfected (control) cells (*** p<0.001, Student᾽s *t* test).

During *in vivo* infections, trophoblasts may secrete proinflammatory mediators not only in response to the pathogen itself but also in response to soluble factors produced by other infected cells, including phagocytes. As *B*. *canis*-infected canine trophoblasts secreted chemoattractants for monocytes (RANTES) and neutrophils (IL-8), and as a histiocytic-neutrophilic infiltrate has been described in *B*. *canis*-infected placentas, we investigated whether factors produced by *B*. *canis*-infected monocytes and neutrophils could influence the production of cytokines and chemokines by trophoblasts. To this end, the later cells were stimulated with conditioned media (CM) from the infected phagocytes. Culture supernatants from these stimulated trophoblasts were harvested at 24 h post-stimulation to analyze the presence of cytokines and chemokines. Levels of these factors already present in the CM (S1 Table) were subtracted in order to determine the secretion specifically elicited by this stimulation. As shown in Fig 3A, the stimulation of trophoblasts with CM from infected monocytes induced a significant increase in IL-8, IL-6 and RANTES secretion as compared to levels found in either trophoblasts stimulated with CM from uninfected monocytes or in unstimulated trophoblasts. Likewise, stimulation with CM from infected neutrophils also induced an increased production of these cytokines in trophoblasts, as levels were significantly higher as compared to levels found in either tropohoblasts stimulated with CM from uninfected neutrophils or in unstimulated trophoblasts (Fig 3B).

Previous studies have shown that human trophoblasts increase their production of IL-8 and RANTES in response to TNF-α [25,26]. Of note, TNF-α levels were significantly increased in CM from *B*. *canis*-infected canine neutrophils and monocytes as compared to their uninfected counterparts (Fig 3C).

### Cytokine response of canine placental explants to *B*. *canis* infection

**Fig 4:**
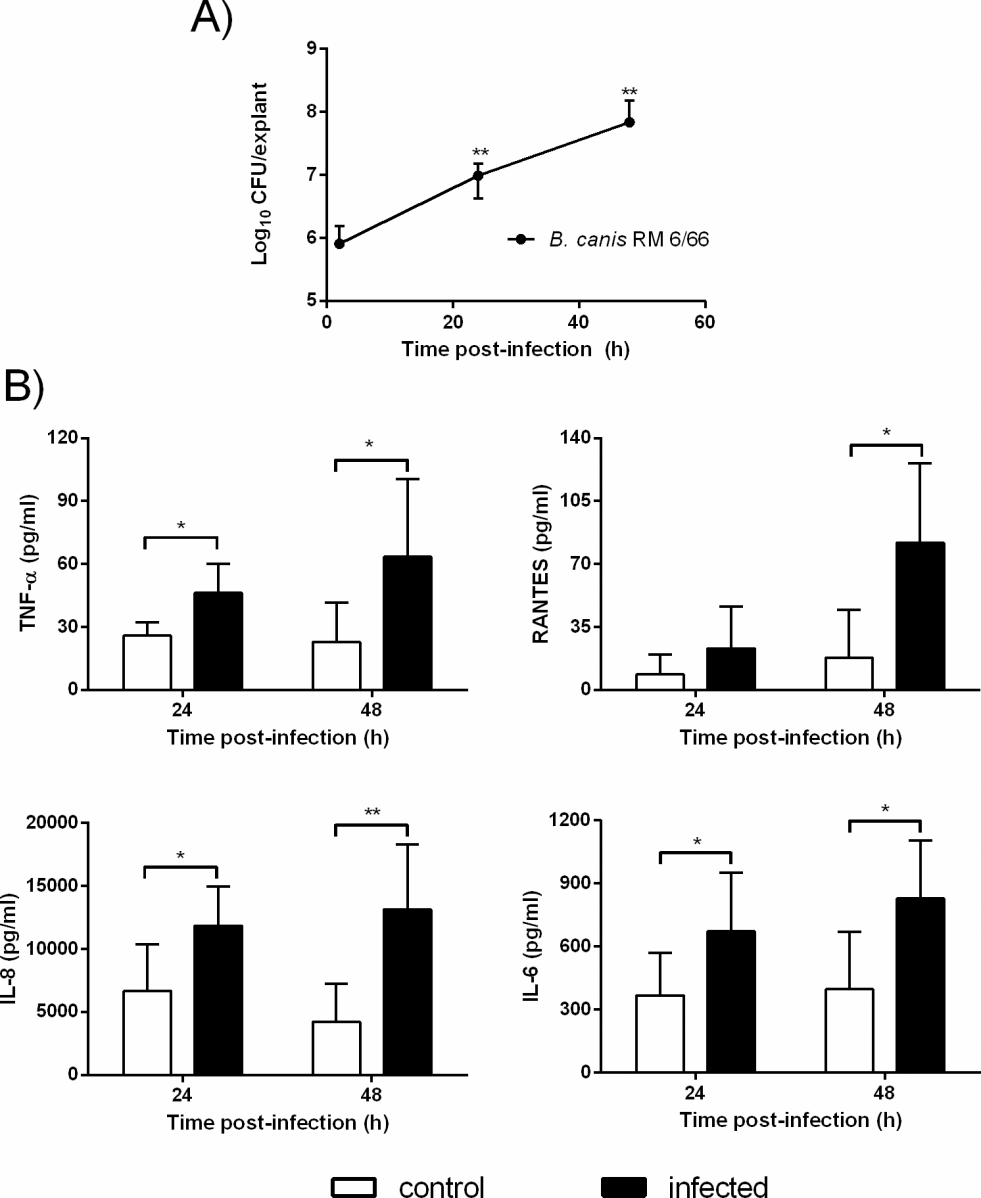
Intracellular bacterial replication and cytokine secretion in canine placental explants infected *ex-vivo* with *B*. *canis*. Explants were obtained from 3 term placentas collected by C-section of healthy dogs, and were infected with 1x10^8^ CFU of *B*. *canis* for 2 h. The explants were washed with PBS and extracellular bacteria were killed by the addition of antibiotics. (A) Explants homogenates were obtained at 2, 24 and 48 h p.i. and plated on TSA to count intracellular CFU. (B) Culture supernatants were collected at 24 and 48 h p.i. to measure TNF-α, RANTES (CCL5), IL-8 and IL-6 secretion. Values are means ± SD of duplicate measurements from three independent experiments. In panel A, asterisks indicate significant differences as compared to 2 h. p.i. (** p<0.01, Mann-Whitney test). In panel B, asterisks indicate significant differences as compared to uninfected (control) cells (* p<0.05; ** p<0.01, Student᾽s *t* test).

The above results suggest that trophoblasts may produce proinflammatory cytokines not only in response to *B*. *canis* infection but also in response to factors produced by *B*. *canis*-infected phagocytes. To evaluate the global inflammatory response of the placental tissue, where these cell interactions may take place in the context of the natural tissue architecture and cellular proportions, canine placental explants were infected with *B*. *canis*, and bacterial replication and cytokine secretion were assessed. As shown in Fig 4A, *B*. *canis* was able to replicate in explants from term canine placentas as intracellular CFU counts increased significantly at 24 and 48h p.i. as compared to 2 h p.i. Cytokine and chemokine analyses revealed a significant increase in TNF-α, RANTES, IL-6 and IL-8 secretion at 24 and/or 48h p.i. in explants infected with *B*. *cani*s as compared to non-infected controls (Fig 4B).

## Discussion

The exact pathophysiological mechanism leading to abortion by *B*. *canis* in pregnant bitches has not been established, but the consistent presence of infiltrates of neutrophils and mononuclear cells in aborted canine placentas [5,8,9] suggests that the inflammatory response may play an important role. Trophoblast cytokine responses are known to contribute to placental inflammation leading to infectious abortion by other pathogens [12–16] a fact that is especially interesting as heavily infected canine trophoblasts are observed in *B*. *canis* abortions. In line with these in vivo observations, the results of the present study confirm that *B*. *canis* is able to infect and replicate in canine trophoblasts. After an initial reduction of viable intracellular bacteria, CFU counts increased sharply between 24 and 48 h p.i. While this study is, to our knowledge, the first one to characterize the kinetics of *B*. *canis* infection in canine trophoblasts (or in any canine cell) it is interesting to note that the results agree with those of a recent study on *B*. *canis* replication in a murine macrophagic cell line and a human epithelial cell line [27]. Also in agreement with that study, the infection did not induce cytotoxicity. It has been shown that, as with other members of the *Brucella* genus, the ability of *B*. *canis* to survive and replicate intracellularly depends on the expression of *virB* genes coding for a type IV secretion system. It is known that the expression of these genes is induced once the bacteria are internalized into cells [28]. Therefore, the initial decline in *B*. *canis* CFU counts observed in canine trophoblasts (this study) and other cells [27] may be related to the lag phase before full *virB* expression.

As mentioned above, in different animal species the cytokine response of trophoblasts to microbial pathogens contributes to placentitis leading to infectious abortion. Cytokine response is in part induced by pathogen-associated molecular patterns (PAMPs) which trigger intracellular responses upon interactions with TLR receptors. Although TLR expression has been well characterized in trophoblasts from different species, including human [29], guinea pig [30] and sheep [14], there are no similar reports on canine trophoblasts. Therefore, we first characterized the cytokine response of primary trophoblasts to different TLR ligands. The increased secretion of IL-8 and RANTES (CCL5) by trophoblasts stimulated with PAM3CK4 and flagellin evidenced the presence of functional TLR1/2 and TLR5 signaling pathways, respectively. In contrast, no response to the TLR4 ligand (LPS) could be detected. Of note, previous studies have shown that the proinflammatory response to *Brucella* antigens relies on the recognition of outer membrane lipoproteins by TLR2 but does not require TLR4 recognition [31].

To evaluate trophoblast specific response to *B*. *canis* we stimulated cells with bacterial antigens (HKBc) and also performed *in vitro* infection. In both cases, caninetrophoblasts responded with an increased secretion of IL-8 and RANTES. As IL-8 is a well known chemoattractant for neutrophils, these results suggest that trophoblasts-derived IL-8 may be involved in the development of the neutrophilic infiltrate usually observed in the placentas of *B*. *canis* abortions. Similarly, RANTES, which is a chemoattractant for a variety of leukocytes into inflammatory sites, may be involved in the generation of the histiocytic infiltrates in *B*. *canis*-infected canine placentas. The increased production of IL-8 by infected canine trophoblasts is in line with previous reports showing an increased expression of IL-8 mRNA in explants of bovine chorioallantoic membranes infected with *B*. *abortus* on their trophoblastic surface [11]. In addition, increased serum levels of RANTES have been associated with *Brucella*-induced abortion in a murine model of pregnancy, although placental levels of this chemokine were not determined [32].

As mentioned above, both neutrophilic and histiocytic infiltrates are observed in canine placentas from *B*. *canis*-induced abortions. This opens the possibility of neutrophil-trophoblast and monocyte-trophoblast interactions through factors secreted by these phagocytes in response to infection. As shown here, factors secreted by *B*. *canis*-infected canine neutrophils induced the secretion of IL-8, IL-6 and RANTES by canine trophoblasts. The same was true for trophoblasts stimulation with factors produced by *B*. *canis*-infected canine monocytes. These findings suggest that, in the context of *B*. *canis* infection, trophoblasts may not only produce proinflammatory cytokines in response to the bacterium but also in response to factors produced by infected monocytes and neutrophils. The overall proinflammatory immune response to *B*. *canis* infection produced in this *in vitro* model of interactions between cells usually found in the placenta was in complete agreement with the increased secretion of IL-8, IL-6, RANTES and TNF-α in canine placental explants infected *ex vivo* with *B*. *canis*. In addition, the fact that TNF-α was produced by infected placental explants but not by *in vitro*-infected canine trophoblasts clearly indicates that non-trophoblastic cells present in explants also responded to *B*. *canis* infection. Phagocytes are the most probable source of this TNF-α response, and our studies showed that monocytes and neutrophils from normal dogs produce this cytokine in response to *B*. *canis*. The infection of phagocytes within explants may also explain why *B*. *canis* CFU increased in explants between 2 and 24 h p.i., whereas the bacterium did not replicate in primary canine trophoblasts *in vitro* during this time frame.

Overall, these results suggest an amplifying mechanism by which canine trophoblasts respond to *B*. *canis* infection with the secretion of IL-8 and RANTES, which attract neutrophils and monocytes to the infection site, and these phagocytes upon contact with the bacterium produce factors that further increase the inflammatory response of trophoblasts. Similar interactions between human trophoblasts and phagocytes in the context of *Brucella abortus* infection have been recently described by us [33].

The nature of the factors produced by infected neutrophils and monocytes that stimulated the cytokine responses in canine trophoblasts is unknown. However, previous studies have shown that human trophoblasts increase their production of IL-8 and RANTES in response to TNF-α [25,26]. The fact that TNF-α was produced by canine neutrophils and monocytes in response to *B*. *canis* infection suggests that this factor may be involved in the induction of IL-8 and RANTES in canine trophoblasts by conditioned media from infected phagocytes.

In summary, this study shows that canine trophoblasts produce proinflammatory cytokines in response to *B*. *canis* infection and/or to stimulation with factors produced by infected monocytes and neutrophils. These cytokines may contribute to placental inflammation leading to abortion in *B*. *canis*-infected pregnant bitches.

## Supporting information

S1 Fig Flow cytometry analysis of CD45+ cells in the trophoblast population isolated from normal canine placentas. Canine trophoblasts isolated from canine placenta were plated in T75 culture bottles and, after 2 weeks in culture, were harvested by trypsin/EDTA treatment. Cells (5x10^5^ per tube) were labeled with either rat IgG2b:FITC antibody (isotype control) (panel A) or rat anti-dog CD45:FITC antibody (panel B) and analyzed by flow citometry.
(TIF)

S1 File Raw data for cytokines, LDH levels and/or CFU counts in canine trophoblasts, phagocytes and explants stimulated or infected with *B*. *canis*. (XLSX)

S1 Table Cytokine levels in conditioned media (CM) from infected canine phagocytes used to stimulate trophoblasts. (DOCX)
