# Supplementary figures and images for: Proinflammatory response of canine trophoblasts to Brucella canis infection
Source: PLoS One. 2017 Oct 16;12(10):e0186561. doi: 10.1371/journal.pone.0186561 (PMC5643107; doi:10.1371/journal.pone.0186561)

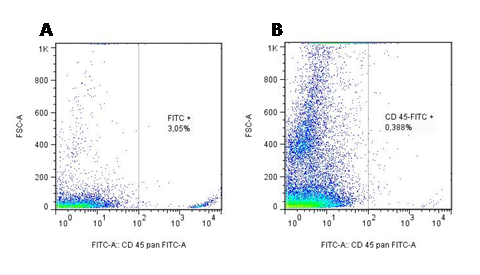

Supplement: S1 Fig — Canine trophoblasts isolated from canine placenta were plated in T75 culture bottles and, after 2 weeks in culture, were harvested by trypsin/EDTA treatment. Cells (5x10^5^ per tube) were labeled with either rat IgG2b:FITC antibody (isotype control) (panel A) or rat anti-dog CD45:FITC antibody (panel B) and analyzed by flow citometry. (TIF) [file pone.0186561.s001.tif]
