# Supplementary material for: Proinflammatory response of canine trophoblasts to Brucella canis infection
Source: PLoS One. 2017 Oct 16;12(10):e0186561. doi: 10.1371/journal.pone.0186561 (PMC5643107; doi:10.1371/journal.pone.0186561)
Supplement: S1 Table — (DOCX) [file pone.0186561.s003.docx]

|  |  |  | Cytokines (pg/ml) | | |
| --- | --- | --- | --- | --- | --- |
|  |  |  | IL-8 | IL-6 | RANTES |
| Monocytes | Dog 1  Dog 2 | Uninfected  Infected  Uninfected  Infected | 24639 ± 347  35592 ±2297  55141 ± 5338  58657 ± 4334 | 454 ± 16  393 ± 7  22 ± 5  120 ± 29 | 232 ± 14  275 ± 10  855 ± 34  1013 ± 31 |
| Neutrophils | Dog 1  Dog 2 | Uninfected  Infected  Uninfected  Infected | 5217 ± 1357  10309 ± 230  522 ± 58  5851 ± 862 | 102 ± 19  119 ± 20  nd  nd | nd  nd  nd  nd |

nd: not detected
